# Predicting product information diffusion for sustainable quality management in industry 4.0: an improved Bass model approach

**DOI:** 10.1038/s41598-025-09129-1

**Published:** 2025-07-17

**Authors:** Zhongya Han, Xiangtang Chen, Kepao Miao, Xiaoxiang Wang, Qinlin Li

**Affiliations:** School of Economics and Management, Wenzhou University of Technology, Wenzhou, 325035 China

**Keywords:** Industry 4.0, Product information diffusion, The interest decay effect, Improved bass model, Mathematics and computing, Applied mathematics, Statistics

## Abstract

In the era of Industry 4.0, accurately predicting the popularity of product information is vital for optimizing marketing strategies and supporting sustainable business growth. Although the classical Bass model has been widely used in diffusion research, it overlooks two critical aspects of modern digital environments: the heterogeneity in user decision-making and the temporal decay of user interest. To address these limitations, we propose an enhanced Bass model that integrates a two-phase diffusion framework. This model accounts for user conversion across heterogeneous information sources and incorporates an interest decay mechanism by relaxing the assumption of constant influence coefficients. We validate the model using movie trailer data from the Weibo platform. The results show that our model significantly outperforms the classical Bass model as well as its power-function and exponential-function variants in predicting information popularity. Further analysis reveals that both internal and external influences decay over time, with internal diffusion also shaped by social pressure. These findings offer valuable insights for improving data-driven diffusion models and enhancing digital marketing effectiveness. Future research may explore model applicability across different product categories and social media platforms.

## Introduction

Product information, encompassing details about product quality and features^1^, plays a critical role in shaping consumer behavior and influencing market performance. In the context of Industry 4.0, the widespread adoption of technologies such as the Internet of Things (IoT), artificial intelligence (AI), and big data analytics has revolutionized how product information is generated, disseminated, and consumed. Social media platforms like Twitter, Sina Weibo, and TikTok have emerged as dominant channels for information diffusion, enabling firms to engage with global audiences rapidly and efficiently. However, this digital transformation also introduces new challenges, particularly in predicting the popularity of product information—an essential task for optimizing marketing strategies and ensuring sustainable business outcomes.

The rapid expansion of information-sharing on social media has prompted firms to increasingly rely on high-exposure accounts—particularly Key Opinion Leaders (KOLs)—to amplify product promotion efforts. Although investment in KOL marketing has grown in recent years, the 2024 China Market Key Opinion Leader (KOL) Marketing White Paper reports a noticeable deceleration. Specifically, the growth rate of KOL marketing investment in China declined to 26% in 2024, representing a 2-percentage-point drop from the previous year. Additionally, the rate of increase in KOL promotion pricing fell by 1.3% points to 13.16%. This slowdown is largely attributed to marketers’ limited ability to accurately forecast the diffusion performance of product information, which in turn weakens confidence in KOL-based strategies. As a result, accurately predicting the popularity of product information has become crucial for developing effective marketing strategies, improving campaign efficiency^2^, and ensuring sustainable resource allocation—especially in the context of emerging formats such as live-streaming e-commerce.

Despite the growing interest in predicting product information popularity, achieving accurate forecasts remains challenging due to three key obstacles. First, historical diffusion data are typically limited. Using historical data for forecasting is efficient and convenient, but the real-time nature and short life cycles of social media content limit the availability and reliability of such data^3^. Second, the sharing process is influenced by a wide range of factors, including content attributes, dissemination context, and source credibility^4^, making the prediction task inherently complex. Third, heterogeneity in user decision-making further complicates diffusion modeling. Information from different sources—such as celebrities versus ordinary users—has varying levels of persuasive power and engagement impact^5^. For example, information from celebrities typically attracts greater user engagement compared to ordinary sources. Moreover, users’ preferences vary significantly across different product information categories^6^.

Many existing approaches to predicting content popularity rely on feature-based machine learning models, which use extracted attributes to train regression or classification algorithms that estimate metrics such as views or shares. However, these models often overlook user-level behavioral heterogeneity and fail to provide insights into the underlying mechanisms that drive information diffusion. Additionally, due to the heavy-tailed nature of popularity distributions on social networks, most models generate probabilistic intervals rather than point predictions^7^. While such interval estimates may capture general trends, they are often inadequate for marketers who require precise forecasts to optimize advertising placements and budget al.location.

The Bass model is a classical framework widely employed in forecasting product adoption, particularly for innovative goods with limited historical data^8,9^. It captures user decision-making by categorizing adopters into two groups: innovators, influenced primarily by external forces such as mass media and promotions, and imitators, driven by internal factors like word-of-mouth and peer influence^10^. While the model has been extensively applied in traditional product diffusion contexts, it faces several limitations when used to model the diffusion of product information. First, it assumes static market potential, neglecting the dynamic nature of information diffusion processes^11^. As information diffuses, users receiving information from different sources gradually become potential sharers, causing the market potential to evolve dynamically. Second, the model presumes external influence remains constant, while internal influence increases monotonically with adopters. In reality, user interest in information typically decays over time, following an exponential function^12^. This interest decay originates from internal information content and external contexts. Consequently, both internal and external influences likely diminish over time. Moreover, increasing sharer numbers may amplify information attractiveness through social pressure^13^, complicating diffusion dynamics further.

Many prior extensions of the Bass model continue to assume a static market potential and fixed influence coefficients, limiting their ability to reflect the dynamic and fast-evolving nature of diffusion on social media platforms. In particular, existing research largely neglects two critical aspects: the heterogeneity of user information sources—such as differences between fan and non-fan audiences—and the temporal decline in user interest. For instance, although Han et al.^14^ introduced external influence decay by modifying the external coefficient, their model did not account for internal interest decay or the dynamic evolution of market potential resulting from heterogeneous user exposure.

To address these gaps, this study extends the classical Bass diffusion model by incorporating two critical but previously underexplored elements in the context of product information diffusion: heterogeneity in information sources and the interest decay effect. We develop a two-phase diffusion framework that captures users’ progression from exposure to decision-making and, ultimately, to sharing behavior. In our model, users are segmented into fans and non-fans based on how they receive information, and we examine how factors such as source type, diffusion context, and content quality influence their sharing behavior. Building on this foundation, we introduce two key enhancements to the Bass model: (1) we redefine market potential as a dynamic function of cumulative sharers to reflect evolving exposure patterns across heterogeneous sources, and (2) we incorporate exponential decay into both internal and external influence coefficients to capture time-sensitive engagement decline. These innovations culminate in a Generalized Improved Bass Model (GBM), which simulates product information diffusion by predicting both the cumulative and unit-time number of shares.

We empirically validate the proposed GBM using movie trailer data collected from Sina Weibo, and benchmark its performance against the classical Bass model (BM), the power-function improved model (PIM), and the exponential-function improved model (EIM) proposed by Han et al.^14^. We further assess the contribution of key model features. The experimental results show that GBM consistently outperforms all baseline models in terms of predictive accuracy. Moreover, both internal and external influence factors exhibit clear temporal decay, and social pressure intensifies internal diffusion. These findings enhance our understanding of product information diffusion patterns on social media and offer actionable insights for optimizing digital marketing strategies in the Industry 4.0 landscape.

This study contributes to the literature in three key ways: (1) It introduces a two-phase diffusion framework that explicitly models user conversion based on heterogeneous information sources (e.g., fans vs. non-fans), thereby enabling dynamic estimation of market potential—addressing a key limitation of existing models that assume static potential and overlook the impact of source heterogeneity. (2) It integrates exponential interest decay into both internal and external influence mechanisms, effectively capturing time-sensitive behavioral shifts and the amplifying effect of social pressure—factors largely ignored in prior models. (3) It proposes a generalized improved Bass model that enhances forecasting accuracy for product information diffusion and extends the classical framework’s applicability to dynamic digital environments.

The remainder of this paper is organized as follows: Sect. 2 reviews the relevant literature. Section 3 presents the construction of the GBM. Section 4 provides empirical evaluations and discussion. Section 5 concludes the study and outlines avenues for future research.

## Related work

Our work involves two research areas: information popularity prediction and the improved Bass model. We review the most pertinent studies in these domains and emphasize the primary distinctions.

### Information popularity prediction

Research on information popularity prediction has predominantly followed two methodological paradigms: feature-based machine learning approaches and behaviorally grounded generative models. Feature-based approaches typically involve extracting various features from textual, temporal, structural, and contextual information and using them to train supervised learning models. For example, Yang et al.^3^ introduced the Named Entity Topic Model (NETM) to capture topic-based textual factors in news popularity growth. Chen and Chang^15^ used metadata such as uploader identity and keywords to predict video popularity using unsupervised learning. Nisa et al.^16^ and Wang et al.^17^ incorporated visual and emotional features for predicting YouTube and social media post popularity, while Xiao et al.^18^ emphasized the role of content, recipient, and source features. These models often employ classification or regression techniques and achieve reasonable performance in identifying likely viral content. While feature-based methods are valuable for capturing surface-level correlations, they suffer from two major limitations. First, most models predict probability intervals or coarse-grained outcomes (e.g., viral or non-viral) rather than precise, time-resolved sharing volumes. Second, they are largely atheoretical—providing little insight into the user decision-making processes that drive diffusion. These limitations reduce their interpretability and applicability in contexts where understanding the dynamics of information spread is critical.

Generative models aim to address these shortcomings by modeling the underlying behavioral or structural processes. Wu et al.^19^ proposed a deep temporal context network incorporating attention mechanisms for time-series popularity prediction. Van et al.^20^ combined content and behavioral data to model news reading dynamics. Chen et al.^21^ used deep learning with attention to reduce noise in user-text-time interactions. Bohra et al.^22^ used tensor decomposition techniques to incorporate historical popularity trajectories. These models provide greater flexibility in capturing time-varying patterns and are more suitable for diffusion-oriented tasks. Nevertheless, most generative models still lack behavioral interpretability. Few explicitly account for interest decay, user heterogeneity, or the cumulative influence of early adopters. Moreover, they often rely on black-box neural architectures, limiting their theoretical transparency.

In contrast, our study adopts a generative approach grounded in user decision theory, offering not only point predictions but also explanatory insight into how interest decay and user-source heterogeneity affect diffusion dynamics.

### Improved Bass model

The Bass model^10^ is a foundational framework for modeling the adoption of innovations, incorporating both internal influence (e.g., social contagion) and external influence (e.g., media exposure). While widely applied in product diffusion research, the model’s core assumptions—particularly its treatment of market potential as static and influence coefficients as constant—limit its effectiveness in capturing the dynamics of behaviorally complex environments such as social media.

To address these limitations, several extensions have been proposed. For example, Mahajan et al.^23^ and Horsky^24^ redefined market potential as a function of price and competitive intensity. Goldenberg et al.^25^ introduced threshold-based contagion mechanisms to model social reinforcement, while Guseo and Guidolin^26^ modeled a time-varying market potential influenced by communication network growth. These enhancements improve the Bass model’s adaptability by relaxing the assumption of a static potential.

Other studies have focused on revising assumptions related to influence coefficients. Bass^27^ for instance, generalized the original model to incorporate marketing and pricing effects. Cosguner and Seetharaman^28^ developed the Bass-Gumbel and Bass-Logit models to capture utility-based adoption behavior, and Wang and Sun^29^ introduced variable influence coefficients that respond to changing environmental conditions. While these efforts enrich the behavioral realism of the model, they have primarily been applied to traditional product sales or pricing strategies.

In contrast, extensions of the Bass model in the context of information diffusion remain limited. Han et al.^14^ proposed a Power-function Improved Model and an Exponential-function Improved Model to account for the decay of external influence. However, their models did not address internal interest decay, the role of social pressure, or the dynamic evolution of market potential shaped by heterogeneous information sources. Our study extends this work in three ways. First, we incorporate exponential decay in both internal and external influence terms, capturing time-dependent disengagement. Second, we introduce a social pressure variable to reflect the social pressure effect. Third, we model market potential as an endogenous function of cumulative adopters, enabling the model to adapt to real-time sharing dynamics. These enhancements offer a more realistic representation of product information diffusion, particularly in social media environments characterized by temporal volatility and user heterogeneity.

### Summary and research gap

In summary, while both feature-based and generative models have improved predictive accuracy, they often lack explanatory depth and fail to reflect the underlying behavioral mechanisms of information diffusion. Similarly, most extensions of the Bass model have been confined to product adoption scenarios and remain ill-equipped to address the complex dynamics of digital content diffusion—especially in environments marked by user heterogeneity, interest volatility, and amplified social influence.

This study addresses these shortcomings by introducing a Generalized Bass Model that systematically integrates a two-phase decision process, user segmentation based on information source (e.g., fan vs. non-fan), exponential interest decay, and dynamic contagion effects. By embedding these behavioral and temporal dimensions, the GBM enhances both the explanatory and forecasting power of the classical Bass framework. This advancement not only fills theoretical gaps in prior diffusion models but also expands their applicability to real-time, high-velocity information environments that typify Industry 4.0 contexts.

## Construction of the generalized improved bass diffusion model

In this section, we first develop a two-phase diffusion framework to accurately represent user decision-making and sharing processes. Building upon this foundation, we propose specific refinements to the classical Bass model, ultimately leading to a GBM. The GBM systematically integrates user segmentation based on information source heterogeneity and incorporates the effect of user interest decay. Finally, we detail the individual components of our model, clearly highlighting their roles in effectively capturing information diffusion dynamics in social media contexts.

### Diffusion process of product information

Product information—encompassing key attributes and features—plays a critical role in shaping users’ decisions to share content. Factors such as publisher reputation, information quality, and dissemination context substantially influence user engagement and sharing behavior^30^. To systematically capture these dynamics, we propose a two-phase diffusion framework that operates at both the individual and group levels.

**Fig. 1:**
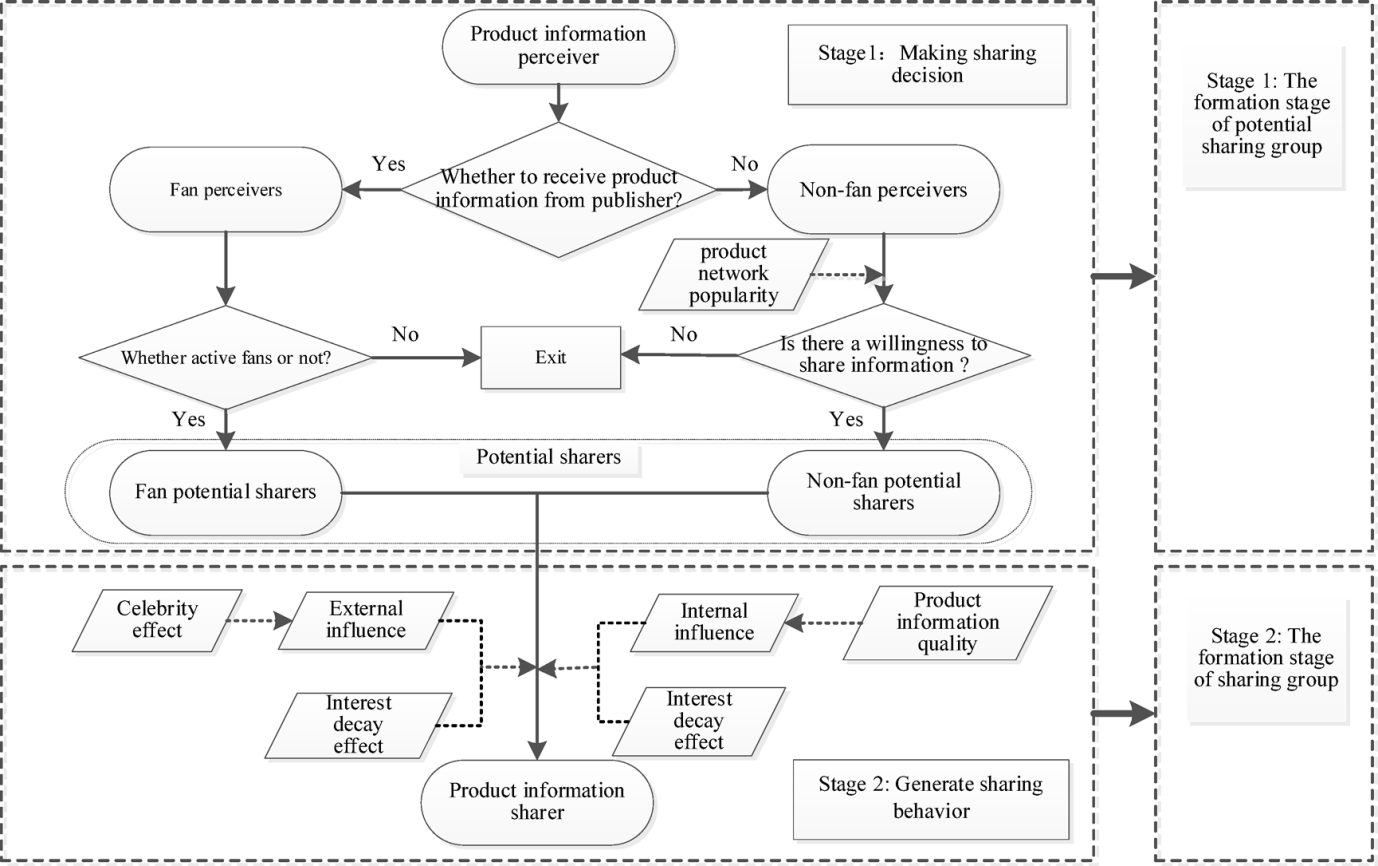
The two-stage sharing model.

**Fig. 2:**
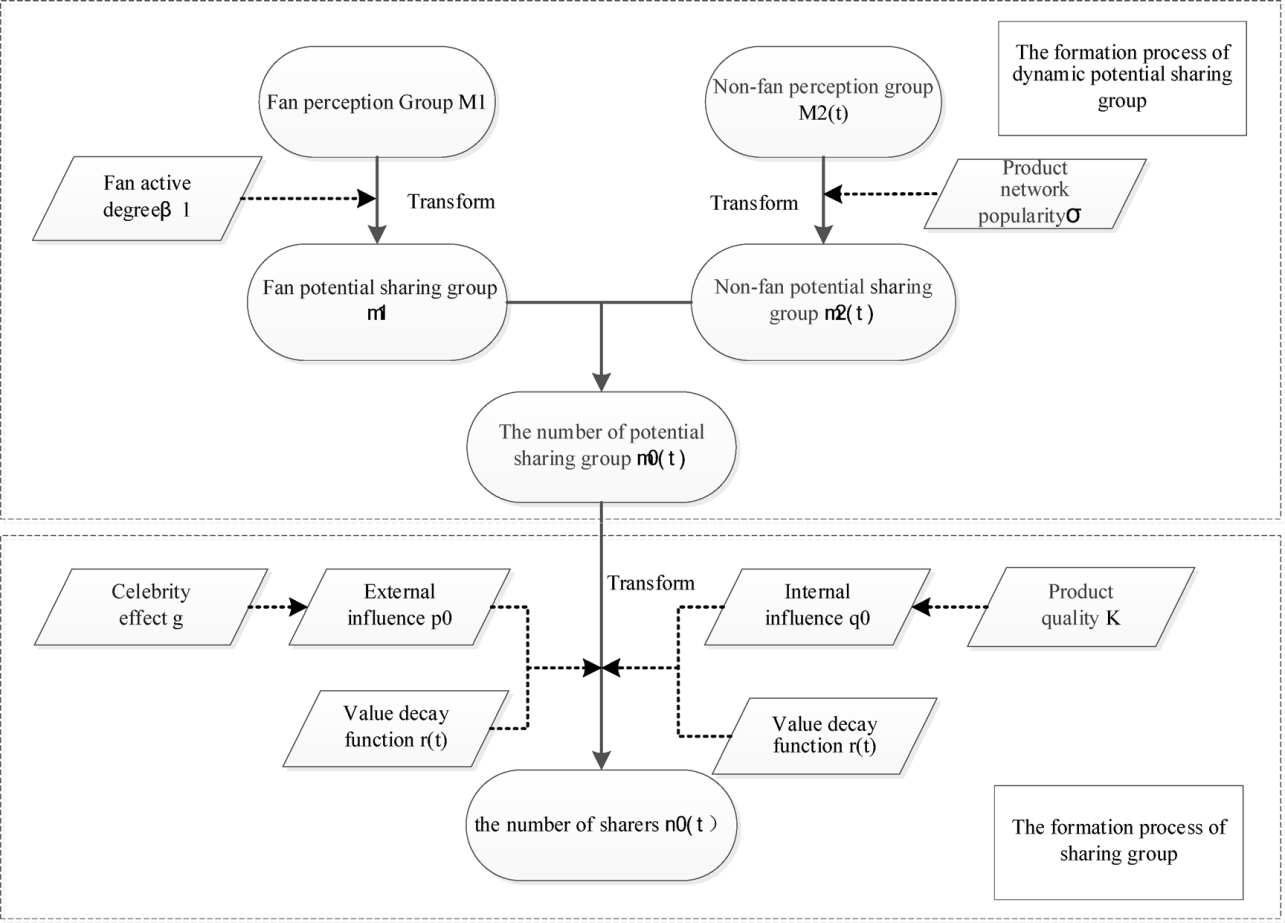
The two-stage diffusion model.

According to the theory of planned behavior, the process from users’ perception of information to sharing information can be divided into two stages: making a sharing decision and performing the sharing action^31^. The decision-making stage involves the process of users from obtaining information to generating sharing intentions, while the action stage involves the process of users from sharing intentions to generating actual sharing behavior. Referencing Fan et al.^32^, we construct a two-stage sharing model and a two-stage diffusion model, as shown in Figs. 1 and 2.

#### The two-stage sharing model

As shown in Fig. 1, the two-stage model for individual users is divided into the decision-making and action stages. In these stages, users who perceive product information first convert into potential sharers in various ways and then into actual sharers. Perceivers are users who have viewed the product information. Potential sharers are those who intend to share the information but have not yet done so. Users do not share product information multiple times.

During the decision-making stage, different types of product information perceivers convert into potential sharers in different ways. Users’ decision processes differ based on the source of the perceived information, which needs to be considered separately. Based on the source of perceived information, perceivers can be categorized as fans or non-fans. Fans are followers of the publisher and receive information directly from them, while non-fans receive information from sharers. When a publisher releases product information, active fans directly become potential sharers, while non-fans may convert into potential sharers under the influence of product network popularity. Active fans refer to fans who often interact with publishers. Product network popularity refers to the attention of all users on the network to the product.

In the action stage, potential sharers convert into actual sharers under the combined influence of internal and external factors. According to social influence theory, the celebrity effect of the publisher corresponds to the external influence in the Bass model, while the quality of the product information corresponds to the internal influence^14^. Fan potential sharers receive product information from the publisher and are mainly influenced by the publisher’s celebrity effect. Non-fan potential sharers receive information from other sharers and are mainly influenced by the product information quality, with this influence increasing as the number of sharers grows. For both groups, the motivation to share declines over time due to interest decay. Specifically, the celebrity effect pertains to the impact that publishers have on users’ sharing behavior, while product information quality concerns the details of the product’s attributes communicated through the shared content.

#### The two-stage diffusion model

In parallel with Figs. 1 and 2 illustrates a two-stage diffusion model from the perspective of user groups. This model depicts the evolution of two key populations: the dynamic potential sharing group and the actual sharing group.

##### The development process of dynamic potential sharing group

**Fig. 3:**
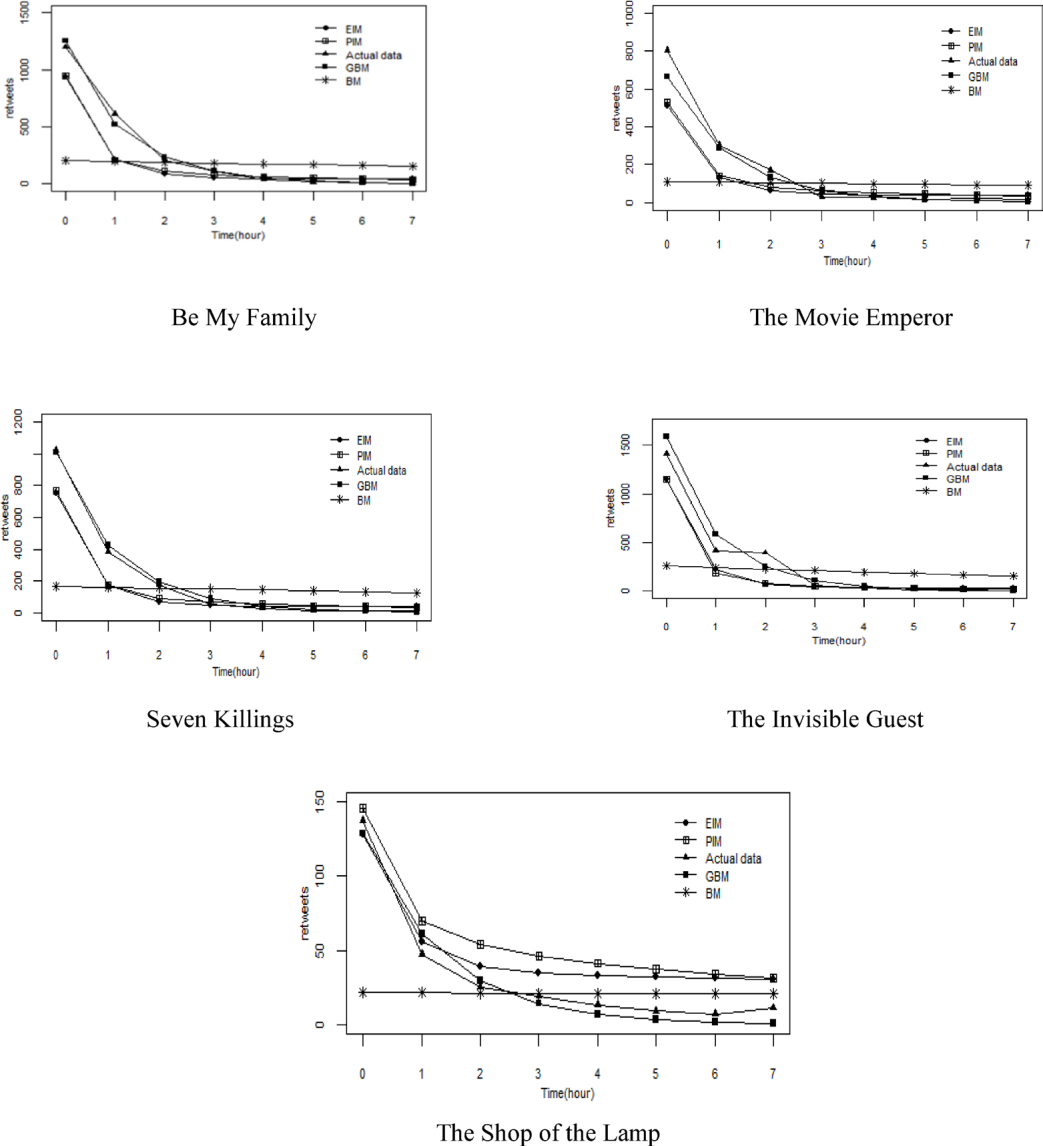
Comparison of sharing times per unit.

Figure 3 demonstrates the transformation process of both non-fan and fan perception groups into potential sharing group, driven by the effects of fan activity and product network popularity. As a modeling simplification, and considering the short diffusion cycle of product information as well as the typically limited change in user status during this period, we assume that users retain their initial status (fan or non-fan) throughout the diffusion process, with no transitions occurring between the two groups.

The fan perception group includes followers who regularly engage with the publisher’s content. Once the publisher shares product information, active fans in this group are likely to form the intention to share it. The shift from the fan perception group ${M_2}(t)$ to the potential fan sharing group δ is influenced by fan activity$(\delta >0)$.The greater the fan activity, the more active fans exist within the fan perception group, leading to an increased number of fan perceiver transitioning into the potential fan sharing group^33^. Therefore, the relationship expression for the potential fan sharing group ${M_2}(t)$ is shown in Eq. (1).

1 $${m_1}={\beta _1}{M_1}$$.

The non-fan perception group acquires information from the sharers of product information. The size of this group grows in proportion to the actual number of sharers. Thus, the size of non-fan perception group${M_2}(t)$ can be defined as the product of the cumulative number of sharers ${N_0}(t)$ and the information spread rate $\varphi$, $(\varphi >0)$. The information spread rate indicates how many unknown individuals can see the product information shared by a single existing sharer. Equation (2) presents the expression of ${M_2}(t)$.

2 $${M_2}(t)=\varphi {N_0}(t)$$.

The non-fan potential sharing group ${m_2}(t)$ originates from the non-fan perception group${M_2}(t)$, with the transformation influenced by the product network popularity $\delta$, $(\delta >0)$. As the product’s network popularity increases, a larger number of non-fan perceivers are converted into non-fan potential sharers. The relationship expression of the non-fan potential sharer group${M_2}(t)$ at time *t* is shown in Eq. (3).

3 $${m_2}(t)=\delta \varphi {N_0}(t)$$.

Let ${\beta _2}=\delta \varphi$. ${\beta _2}$ denotes the sharing rate for product information, meaning that each unit of information shared by the original sharer will be shared by ${\beta _2}$ users, ${\beta _2}>0$. Equation (3) can then be rearranged as Eq. (4).

4 $${m_2}(t)={\beta _2}{N_0}(t)$$.

The fan potential sharing group and the non-fan potential sharing group together form the potential sharing group for product information. It is assumed that there is no mutual conversion between these two groups during the product information diffusion cycle. Equation (5) provides the expression of potential sharers ${m_0}(t)$ at time *t*.

5 $${m_0}(t)={m_1}+{m_2}(t)={\beta _1}{M_1}+{\beta _2}{N_0}(t)$$.

##### The development process of sharing group

The potential sharing group of product information transforms into the actual sharing group under the combined influence of external and internal factors.

The publisher significantly impacts product information diffusion^34^. The celebrity effect describes the degree to which publishers affect user sharing behavior. As the celebrity effect becomes more pronounced, its external impact on the dissemination of product information increases. Consequently, the external influence of the Bass model can be represented as a function of the celebrity effect, as outlined in Eq. (6).

6 $${p_0}={\gamma _1}g$$.

In the Equation, ${p_0}$ indicates the external influence of the product information diffusion, *g* denotes the celebrity effect, and ${\gamma _1}$ is the coefficient of the celebrity effect’s influence, ${\gamma _1}>0$.

The quality of information significantly impacts information diffusion^35^. The quality of product information refers to the details of the product’s attributes perceived by users. Given that product reputation serves as an indicator of product quality, the quality of the product information can be modeled as a function of this reputation. The internal influence of the Bass model can be represented as a function of product information quality. This is detailed in Eq. (7).

7 $${q_0}={\gamma _2}K$$.

In the Equation, ${q_0}$ indicates the internal influence of the product information diffusion, *K* denotes the product information quality, and ${\gamma _2}$ is the coefficient of the impact of product information quality, ${\gamma _2}>0$.

The interest that users have in sharing product information declines nonlinearly as time progresses^36^. According to Radvansky et al.^13^ and Han et al.^14^, the effect of interest decay can be represented by an information value decay function, which follows an exponential decay pattern. Therefore, the interest decay function is assumed to exhibit an exponential decline over time, as shown in Eq. (8).

8 $$r(t)={e^{ - \alpha t}}$$.

In this Equation, $r(t)$ represents the information value decay function, and *α* is the interest decay parameter, $\alpha >0$.

The influence of product word-of-mouth on users’ purchasing behavior has a social pressure effect^29^. Considering the social pressure effect by the group that has shared the product information on the group that has not, the number of sharers ${n_0}(t)$ at time *t* can be expressed by Eq. (9).

9 $${n_0}(t)=r(t)({\gamma _1}g+{\gamma _2}KF)({m_0}(t) - {N_0}(t))$$.

In this Equation, *F* denotes the ratio of users who have shared the information to the total user population.

### Proposed improved Bass model

Two main factors drive product information diffusion on social media platforms: the product information quality and the celebrity effect of the publisher, corresponding to internal and external influences in the Bass model. However, the diffusion process is more complex—potential sharers are affected by the number of actual sharers, and users’ willingness to share diminishes over time. Consequently, the Bass model needs refinement. Building on the analysis in Sect. 3.1, we have expanded the model’s assumptions.

#### H1

With the rise in actual sharers, the number of potential sharers also grows.

#### H2

The appeal of product information to users grows as the frequency of sharing increases.

#### H3

The interest of potential sharers in sharing the product information decays over time.

According to Horsky (1990), Eqs. (10) and (11) are used to represent the number of potential sharers and the likelihood of them sharing information at time *t*, respectively.

10 $${m_0}(t)={m_0}(0)+{\beta _2}{N_0}(t)$$

11 $${P_0}(t)=\frac{{{f_0}(t)}}{{1 - {F_0}(t)}}=({p_0}+{q_0}{F_0}(t)){e^{ - at}}$$.

In these Equations, ${m_0}(0)$ represents the number of users with the intention to share the product information at the initial release. ${N_0}(t)$ indicates the cumulative number of product information sharers at time *t*, ${N_0}(t)={m_0}(t){F_0}(t)$.${f_0}({\text{t}})$ denotes the speed of product information sharing at time *t*. ${F_0}(t)$ is the ratio of cumulative sharers to potential sharers at time *t*. ${P_0}(t)$ reflects the probability that a potential sharer who has not yet shared the product information will share it at time *t*.

When $F(0)=0$, the differential Eq. (11) can be solved, yielding the analytical solution shown in Eq. (12).

12 $${F_0}(t)=\frac{{{p_0}\left[ {{e^{\frac{{{p_0}+{q_0}}}{\alpha }(1 - {e^{ - \alpha t}})}} - 1} \right]}}{{{q_0}+{p_0}{e^{\frac{{{p_0}+{q_0}}}{\alpha }(1 - {e^{ - \alpha t}})}}}}$$.

Equations (10) and (12) can be combined to calculate the cumulative number of sharers *N*_o_(t) at time *t*, which is detailed in Eq. (13).

13 $${N_0}(t)=\frac{{{m_0}(0)[{e^{\frac{{{p_0}+{q_0}}}{\alpha }(1 - {e^{ - \alpha t}})}} - 1]}}{{\frac{{{q_0}}}{{{p_0}}}+{\beta _2}+{p_0}{e^{\frac{{{p_0}+{q_0}}}{\alpha }(1 - {e^{ - \alpha t}})}}(1 - {\beta _2})}}$$

Equations (10)–(13) allow us to derive both the number of potential sharers ${m_0}(t)$and the number of actual sharers ${n_0}(t)$at time *t*.These results are presented in Eqs. (14) and (15).

14 $${n_0}(t)=\frac{{{p_0}{m_0}(0){{({p_0}+{q_0})}^2}{e^{\frac{{{p_0}+{q_0}}}{\alpha }(1 - {e^{ - \alpha t}}) - at}}}}{{({q_0}+{p_0}{e^{\frac{{{p_0}+{q_0}}}{\alpha }(1 - {e^{ - \alpha t}})}})({\beta _2}{p_0}+{q_0}+(1 - {\beta _2}){p_0}{e^{\frac{{{p_0}+{q_0}}}{\alpha }(1 - {e^{ - \alpha t}})}})}}$$

15 $${m_0}(t)={\beta _1}{M_1}\left[ {1+\frac{{{\beta _2}[{e^{\frac{{{\gamma _1}g+{\gamma _2}K}}{\alpha }(1 - {e^{ - \alpha t}})}} - 1]}}{{\frac{{{\gamma _2}K}}{{{\gamma _1}g}}+{\beta _2}+{\gamma _1}g{e^{\frac{{{\gamma _1}g+{\gamma _2}K}}{\alpha }(1 - {e^{ - \alpha t}})}}(1 - {\beta _2})}}} \right]$$.

### The generalized improved Bass diffusion model

We integrated Eq. (5) to (14) to derive a generalized improved Bass diffusion model that accounts for user decision processes and interest decay effects, as shown in Eq. (16).

16 $${n_0}(t)=\frac{{{\gamma _1}g{\beta _1}{M_1}{{({\gamma _1}g+{\gamma _2}K)}^2}{e^{\frac{{{\gamma _1}g+{\gamma _2}K}}{\alpha }(1 - {e^{ - \alpha t}}) - at}}}}{{({\gamma _2}K+{\gamma _1}g{e^{\frac{{{\gamma _1}g+{\gamma _2}K}}{\alpha }(1 - {e^{ - \alpha t}})}})({\beta _2}{\gamma _1}g+{\gamma _2}K+(1 - {\beta _2}){\gamma _1}g{e^{\frac{{{\gamma _1}g+{\gamma _2}K}}{\alpha }(1 - {e^{ - \alpha t}})}})}}$$.

As shown in Eq. (16), ${n_0}(t)$ is a function of time *t* that first increases and then decreases. Specifically, at the initial stage, the number of shares per unit time increases with time, but subsequently decreases as time progresses. This mathematical structure ensures that our model possesses greater flexibility compared to the Bass model.

## Model performance evaluation

We empirically evaluate the performance of the proposed model using movie trailer data from Sina Movie, the official movie promotion platform on Sina Weibo, which has a substantial follower base. To ensure consistency and comparability with Han et al.^14^, we used a similar dataset and benchmarked the proposed model against alternative models.

### Data collection and processing

Using Python, we collected sharing data for movie trailers posted by Sina Movie during May and June 2024. After removing incomplete and anomalous entries, we retained a clean dataset comprising 90 movie trailers. To account for peak user activity and limit the effects of repeated sharing behavior, only retweets occurring between 7:00 AM and 12:00 PM were included in the analysis. Given that the typical diffusion cycle for trailers spans 48–60 h, we constrained the observation window to the first 62 h post-release to minimize prediction bias. Word-of-mouth and popularity indicators were collected from Sina Entertainment and Weibo Hot Search, respectively. Of the 90 samples, 85 were used for training and the remaining 5 for testing.

### Variable selection

#### Variables for the potential sharing group

Considering that user activity levels fluctuate over time^37^. To minimize prediction errors, we defined the size of the fan perception group as the maximum sharing number of trailers made by the publisher in the past week, as shown in Eq. (17).

17 $${M_1}=\hbox{max} \{ {b_j}\} ,j \in \{ 1,{n_0}\}$$.

In this equation, ${n_0}$ denotes the total count of trailers released by the publisher in the past week, while ${b_j}$ indicates the number of sharing for the *j*-th trailer.

Non-fan-perceived group is transformed into potential sharing groups under the influence of product network popularity. The product network popularity refers to the attention that a product receives from network users. Since Sina Hot Search can only display up to 12,000 Weibo posts, the total number of sharing of movie-related posts on the network in the 4 h before the trailer release is selected as the product network popularity. This is normalized, as shown in Eq. (18).

18 $$\delta =\frac{{\sum\limits_{{j=1}}^{{{n_1}}} {{d_j}} }}{{12000}}$$.

In this equation, ${n_1}$ indicates the total count of Weibo posts related to the movie in the 4 h preceding the trailer release, and ${d_j}$ is the sharing number of the *j*-th post.

#### Variables for the sharing group

The celebrity effect refers to the impact of the publisher on user sharing behavior. The celebrity effect of the same publisher varies for different product information^5^, and it fluctuates over time^18^. Therefore, the publisher’s celebrity effect is measured by the average number of shares for trailers released by Sina Movies in the past week, which is normalized as depicted in Eq. (19). According to Kerouacian^38^, the quality of the trailer is directly correlated with the reputation of the director and leading actors, with the specific calculation shown in Eq. (20).

19 $$g=\frac{{\sum\limits_{{j=1}}^{{{n_2}}} {{e_j}} }}{{1000{n_2}}}$$

20 $$K=\frac{{{C_1}+{C_2}+I}}{{300}}$$.

In this equation, ${C_1}$, ${C_2}$, and *I* represent the reputations of the male lead, female lead, and director, respectively. If there are multiple male or female leads in the trailer, select the highest-reputation actors.

### Evaluation of model predictive performance

In this section, we evaluated the predictive performance of the GBM model and compared the results with the comparison model. Section 4.3.1 outlines the parameter estimation for GBM, EIM, PIM, and BM models based on the training dataset, followed by the prediction of trailer sharing times in the test set using the obtained parameters. In Sect. 4.3.2, we evaluate the models based on their goodness of fit, prediction accuracy, and robustness. Section 4.3.3 and 4.3.4 focus on comparing the predicted potential sharing times and analyzing both the internal and external influence coefficients.

#### Comparison of estimated parameters

To facilitate the estimation of the parameters in Eq. (16), we organized the trailer data into 4-hour intervals, and got 720 data points. We constructed the objective function $\min (\sum\limits_{{{\text{i}} = 1}}^{k} {\sum\limits_{{t_{i} = 0}}^{{T_{{\text{i}}} }} {(n_{i} (t_{i} ) - \hat{n}_{i} (t_{i} ))} } ^{2} )$, where *k* is the total count of trailers, ${T_i}$ denotes the lifecycle of the i-th trailer, $\hat{n}_{i} (t_{i} )$ indicates the predicted sharing times of trailer *i* at time *t*, and ${n_i}({t_i})$ is the actual sharing times of trailer *i* at time *t*. We optimized the objective function within the training set, and conducted a significance test of the parameters using the asymptotic F-distribution of nonlinear regression. The results are shown in Table 1.

**Table 1:** Parameter Estimation results and significance test.

Model	parameters	${\beta _1}$	$\varphi$	${\gamma _1}$	${\gamma _2}$	$\alpha$
GBM	estimated value	0.61100	0.11200	0.60200	0.00498	0.72000
	p-value	0.00***	0.00***	0.00***	0.00***	0.00***

As presented in Table 1, fan activity plays a crucial role in the diffusion of movie trailers. The high fan activity value implies a high conversion rate from fan perception group to potential sharing group, aligning with real-world observations. Users who share trailers significantly contribute to their diffusion, with the number of potential non-fan sharers increasing in direct proportion to the number of active sharers. The celebrity effect also significantly impacts the diffusion process of trailers, with the publisher’s influence being directly proportional to the external impact on trailer diffusion. Furthermore, the quality of information significantly affects trailer diffusion, where higher information quality leads to a stronger internal impact on trailer diffusion. Finally, the interest decay parameter also has a significant influence on trailer diffusion, as users’ sharing interest in movie trailers decreases over time.

#### Comparison of model prediction results

Based on the estimated parameters, we predicted the sharing number of five movie trailers, and compared the prediction results with those from the Bass model, EIM, and PIM. The comparison results are presented in Tables 2 and 3, and Fig. 3. The parameters for the comparison models were obtained using the same method as our model.

**Table 2:** The goodness of fit and robustness.

Trailer	Model	${R^2}$	RMSE
Be My Family	BM	0.9597	30.50
	GBM	0.9615	27.97
	PIM	0.8523	52.31
	EIM	0.8341	49.43
The Movie Emperor	BM	0.6183	105.70
	GBM	0.9121	36.77
	PIM	0.7834	66.74
	EIM	0.7767	63.29
Seven Killings	BM	0.5078	158.60
	GBM	0.9974	21.84
	PIM	0.8113	57.37
	EIM	0.8031	54.57
The Invisible Guest	BM	0.4622	161.88
	GBM	0.9952	25.46
	PIM	0.8114	58.94
	EIM	0.8052	58.23
The Shop of the Lamp	BM	0.4549	106.99
	GBM	0.9889	12.48
	PIM	0.8543	57.67
	EIM	0.8137	51.39

Table 2 presents the goodness of fit and robustness of the four models. As shown in Table 2, our model outperforms the BM, EIM, and PIM in terms of ${R^2}$ and root mean square error (RMSE).

**Table 3:** Comparison of cumulative sharing quantity.

Model	Be My Family		The Movie Emperor		Seven Killing		The Invisible Guest		The Shop of the Lamp	
	Predicted value	Relative error	Predicted value	Relative error	Predicted value	Relative error	Predicted value	Relative error	Predicted value	Relative error
BM	1530	19.2%	763	46.4%	1538	11.4%	1739	24.0%	198	36.7%
GBM	1929	1.8%	1382	-2.9%	1761	1.1%	2376	3.8%	321	2.6%
EIM	1732	-8.5%	875	38.5%	1178	32.1%	1387	39.4%	453	44.7%
PIM	1701	10.1%	861	39.5%	1197	31.0%	1365	40.4%	406	29.7%
Actual value	1893		1423		1735		2289		313	

**Fig. 4:**
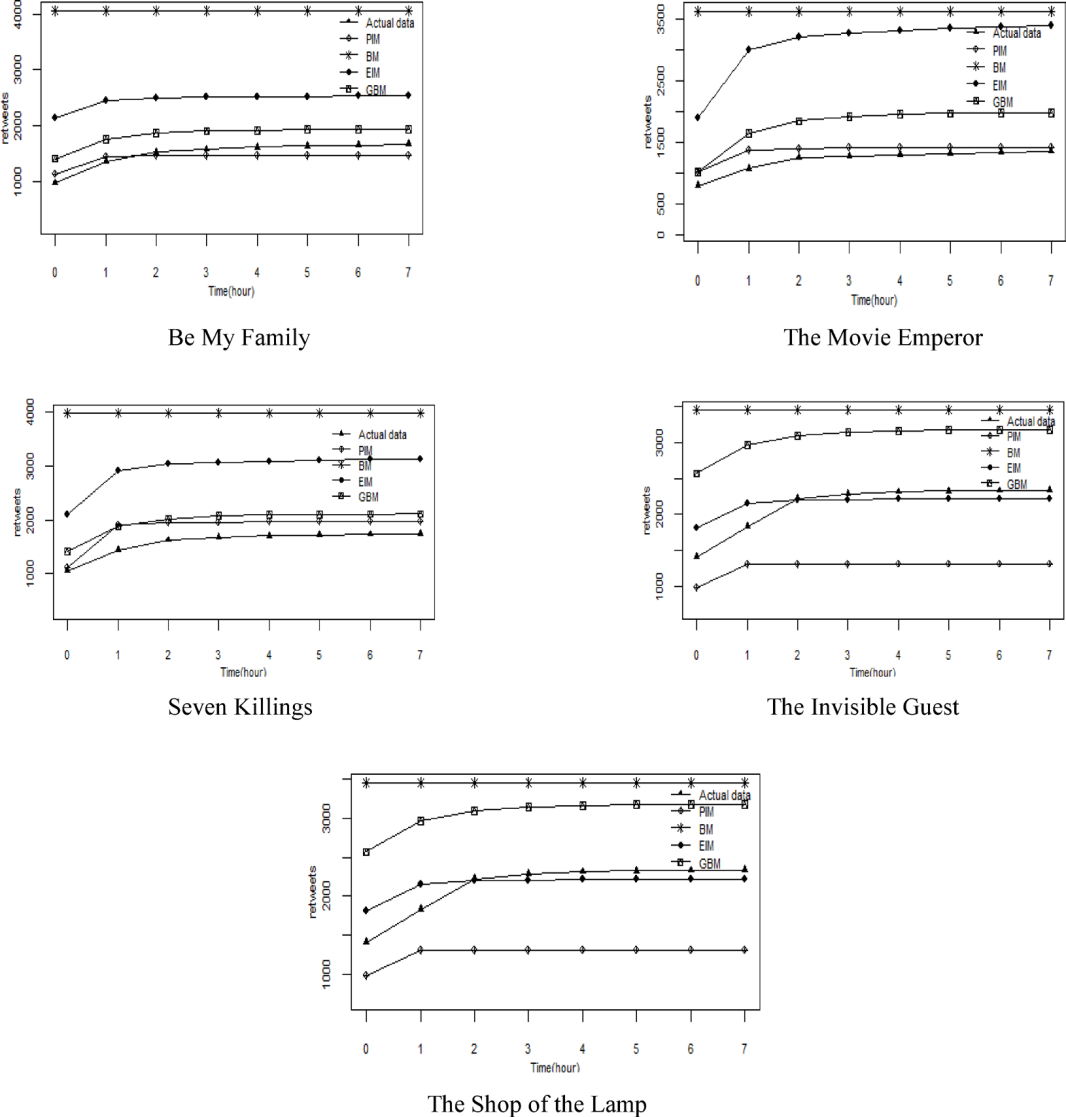
Comparison of the potential sharing number.

Figure 3; Table 3 show the unit time sharing volume and cumulative sharing volume of test set trailers predicted by four models, respectively. As shown in Fig. 3, our model’s predictions for the unit time sharing volume are closer to the actual sharing volume than those of the comparison models, which better reflect the changing trend of movie trailer sharing volume on the social media platform. As shown in Fig. 4, our model’s accuracy in predicting cumulative sharing volume surpasses that of the comparison models.

#### Comparison of the potential sharing quantity

Figure 4 compares the potential sharing number of test set trailers predicted by four models. As shown in the figure, the Bass model’s prediction shows a constant potential sharing number, which is notably lower than the actual sharing figures. In contrast, our model predicts a dynamic potential sharing number that increases over time. Initially, the growth rate is rapid, then it gradually slows as it approaches the actual sharing numbers. The growth rate of dynamic potential sharing number is positively correlated with the growth rate of actual cumulative sharing times. The more actual sharing number generated per unit time, the greater the growth rate of dynamic potential sharing number. This result is also consistent with reality.

Table 4 provides a comparison of the potential sharing numbers forecasted by the four models and the actual cumulative sharing numbers. It is evident that the Bass model predicts a much higher potential sharing number than what was actually observed, whereas the PIM model offers predictions that are more closely aligned with the actual sharing numbers.

**Table 4:** Comparison of the potential sharing number.

Model	Be My Family		The Movie Emperor		Seven Killing		The Invisible Guest		The Shop of the Lamp	
	Predicted value	Relative error	Predicted value	Relative error	Predicted value	Relative error	Predicted value	Relative error	Predicted value	Relative error
BM	4037	113.3%	3563	150.4%	4018	131.6%	3439	50.2%	3708	1084.7%
GBM	1986	4.9%	1951	37.1%	1982	14.2%	3016	31.8%	1091	248.6%
EIM	2563	35.4%	3372	137.2%	3078	77.4%	2197	-4.1%	2973	849.8%
PIM	1451	23.3%	1481	4.1%	1917	10.5%	1365	40.3%	1085	246.6%
Actual value	1893		1423		1735		2289		313	

#### Comparison of the influence coefficients

Table 5 shows the internal and external influence coefficients for the four models. As shown, the coefficients for all models across five trailers remain within expected ranges. The external influence coefficients are significantly higher than the internal coefficients, indicating that potential sharing users are primarily influenced by the publisher’s celebrity effect. This finding aligns with how product information spreads on social media platforms in reality.

**Table 5:** Comparison of the internal and external coefficients.

Trailer	GBM		BM		EIM		PIM	
	q_0_	*p*_0_	q	*p*	q	*p*	q	*p*
Be My Family	0.00384	0.57830	0.00077	0.52625	0.0291	0.2751	0.0793	0.5121
The Movie Emperor	0.00391	0.65940	0.00078	0.60005	0.0269	0.2837	0.0821	0.5433
Seven Killing	0.00388	0.72570	0.00078	0.66039	0.0271	0.4075	0.0831	0.4792
The Invisible Guest	0.00400	0.84000	0.00081	0.76440	0.0289	0.3267	0.07810	0.6274
The Shop of the Lamp	0.00400	0.4735	0.00081	0.43089	0.0287	0.3712	0.0813	0.7247

#### Summary of comparative results

A comparative evaluation based on goodness-of-fit, robustness, forecasting accuracy, estimated potential shares, and influence coefficients reveals that the proposed GBM outperforms the comparison models in both predictive performance and stability. Specifically, in terms of model fit and robustness, the GBM achieves higher goodness-of-fit and lower RMSE values compared to the baseline Bass Model (BM) and the PIM and EIM proposed by Han et al. With respect to forecasting accuracy, the time-series curve of predicted sharing volume per unit time generated by the GBM aligns more closely with the actual diffusion trajectory of the trailer, while its cumulative share predictions exhibit a higher degree of agreement with observed values than those of the comparison models. In the estimation of potential sharing volume, the GBM slightly overestimates the actual number of shares; however, its estimates increase consistently with the actual sharing volume, thereby more accurately reflecting the progressive nature of real-world product information diffusion. Finally, regarding influence coefficients, all four models report higher external than internal influence coefficients, which is consistent with empirical observations and reinforces the dominant role of external factors in driving early-stage diffusion.

### Sensitivity analysis of model parameters

To evaluate the robustness of the GBM, we conducted a sensitivity analysis on key parameters, including fan activity level, celebrity effect coefficient, and interest decay rate. The analysis was performed using data from 90 movie trailers, where each key parameter was independently varied by 10% and 20%, while holding other parameters constant.

As shown in Table 6, the predictive performance of the GBM is robust to changes in the interest decay rate and fan activity level, with only minor fluctuations in RMSE observed under 10% and 20% perturbations. However, the model exhibits higher sensitivity to variations in the celebrity effect coefficient. In particular, increasing the celebrity effect by 10% or 20% leads to a significant rise in RMSE, indicating that the dynamics of external influence have a substantial impact on diffusion predictability. This result is consistent with real-world observations, where sudden increases in external promotional forces (e.g., celebrity endorsements) often lead to explosive diffusion patterns that are difficult to capture with standard modeling assumptions. Future research could explore the development of adaptive external influence models to enhance predictive stability under varying external conditions.

**Table 6:** Sensitivity analysis results.

Parameter	Change (%)	Overall RMSE
Interest decay rate *α*	+ 20	45.23
Interest decay rate *α*	+ 10	44.96
Interest decay rate *α*	− 10	45.21
Interest decay rate *α*	− 20	46.12
Fan activity level ${\beta _1}$	+ 20	44.31
Fan activity level ${\beta _1}$	+ 10	44.83
Fan activity level ${\beta _1}$	− 10	45.13
Fan activity level ${\beta _1}$	− 20	45.38
Celebrity effect ${\gamma _1}$	+ 20	56.67
Celebrity effect ${\gamma _1}$	+ 10	53.91
Celebrity effect ${\gamma _1}$	− 10	48.39
Celebrity effect ${\gamma _1}$	− 20	44.83

### Out-of-sample validation

To further evaluate the predictive capability of the GBM, we conducted an out-of-sample validation using five representative movie trailers previously analyzed in Sect. 4.3. For each sample, the first 70% of the time-series data was used as the training set, while the remaining 30% served as the test set to assess the model’s ability to forecast future diffusion trends. To ensure parameter estimation accuracy, we re-collected the data using a time interval of three hours.

**Table 7:** Out-of-sample validation.

Model	Be My Family	The Movie Emperor	Seven Killing	The Invisible Guest	The Shop of the Lamp
	RMSE	RMSE	RMSE	RMSE	RMSE
GBM	16.31	21.48	13.15	16.63	8.07

The results are presented in Table 7. As shown, the GBM achieved relatively low RMSE values across all five samples, which are noticeably lower than those reported in Table 2. This is expected, as Table 2 focused on pre-release prediction scenarios using only pre-launch data, whereas Table 7 leverages observed historical diffusion for parameter fitting. These results demonstrate that the GBM exhibits strong predictive validity not only under launch-time uncertainty, but also when deployed in early-stage diffusion monitoring tasks.

### Discussion

Our proposed model demonstrates superior performance in terms of model fit, robustness, and predictive accuracy compared to existing models. The diffusion trajectory of product information typically exhibits an asymmetric pattern, characterized by rapid diffusion immediately after initial release, followed by a swift decline^35^. According to Rogers^39^, diffusion encompasses the communication process by which an innovation spreads among members of a social system over time. Researchers have generally conceptualized the diffusion trajectory as an S-shaped curve representing cumulative adopters throughout an innovation’s lifecycle^40,41^. Previous studies indicated that the classical Bass Model performs well for durable goods diffusion but is less effective in capturing the asymmetric diffusion characteristics of high-tech products or digital content^42^. Guseo et al.^43^ enhanced the model by incorporating a shock function to quantify marketing and promotional impacts, achieving improved accuracy for asymmetric diffusion scenarios. Subsequently, Han et al.^14^ extended the Bass Model by incorporating interest decay into the external influence coefficient, effectively capturing the asymmetry in product information diffusion. Building on their approach, we further expanded the model by integrating interest decay into both internal and external influence coefficients and accounting for social pressure effects. This comprehensive enhancement allows our model to capture more accurately the asymmetric nature of information diffusion curves, resulting in superior explanatory power and prediction accuracy compared to the EIM model.

It is worth noting that the prediction performance of our model is better than that of the BM, PIM and EIM, but the potential sharing volume predicted by PIM is closer to the actual value. Unlike methods that adjust diffusion dynamics solely by altering residual market potential^24^ or modifying the internal influence component^25^, our model dynamically integrates market potential as a function of cumulative sharers. This approach aligns closely with that of Guseo and Guidolin^26^, who conceptualized market potential as evolving dynamically with adopter accumulation. However, whereas their work assumes user homogeneity, we extend this framework by explicitly considering heterogeneity among users based on information source—distinguishing between fan and non-fan users influenced by distinct factors. Consequently, although all models (EIM, PIM, and ours) demonstrate potential sharer estimates increasing alongside cumulative sharing, the EIM and PIM generally underestimate actual cumulative sharing numbers. In practice, not all potential sharers transition into actual sharers due to interest decay, resulting in discrepancies between these estimates and real diffusion outcomes^12^. Our model thus provides more realistic and reliable forecasts of sharing dynamics by explicitly capturing the gap caused by interest decay.

In scenarios lacking historical sales data, traditional Bass-based models become difficult to apply directly. To address this limitation, we employed the discrete-choice survey approach introduced by Cosguner and Seetharaman^28^, enabling our model to estimate parameters from comparable historical data. Consequently, our model provides accurate predictions of information popularity even before its actual dissemination. Additionally, by explicitly modeling user decision-making processes based on information sources, our study elucidates the mechanisms through which external (publisher celebrity effect) and internal (content quality, social influence) factors jointly shape diffusion outcomes. Our findings confirm that both internal and external influences on diffusion are subject to interest decay, and that existing sharers partially mitigate this decay through social pressure. This phenomenon aligns closely with memory decay theory^13^.

Compared to baseline models, the GBM provides several distinct advantages. First, by embedding interest decay into both external and internal influences and introducing social pressure as an endogenous mechanism, our model significantly enhances explanatory power and forecasting accuracy, particularly in asymmetric diffusion contexts typical of digital environments. Second, explicitly modeling dynamic market potential as a function of cumulative sharers and differentiating user segments (fan vs. non-fan) results in more accurate and realistic predictions of actual diffusion trajectories. Third, by calibrating parameters using historical diffusion data from analogous scenarios, our model enables proactive forecasting prior to product information release. Despite these advantages, several limitations merit acknowledgment. Introducing additional parameters (e.g., decay rate, fan activity, celebrity effect coefficient) may elevate the risk of overfitting in practical applications. Furthermore, validation conducted solely on Weibo data necessitates further cross-platform evaluations to verify generalizability.

Our research contributes meaningfully to the advancement of diffusion models and their practical applications. By accurately predicting both cumulative and instantaneous sharing volumes, our model offers actionable decision-making insights for marketers in selecting publishers, optimizing advertising budgets, and timing content release strategies. Moreover, our approach provides valuable guidelines for high-traffic influencers and KOLs regarding optimal posting schedules and advertising pricing. Collectively, these advancements facilitate more precise diffusion modeling and support the sustainable management of digital marketing practices in Industry 4.0 contexts.

## Conclusion

In the era of Industry 4.0, accurately predicting the popularity of product information has become essential for designing effective marketing strategies and supporting sustainable business growth. While the classical Bass model remains a foundational framework for modeling diffusion processes, its assumptions of static market potential and constant influence coefficients limit its ability to explain the dynamic, user-driven nature of information sharing in social media environments. To address these limitations, this study proposes a GBM that incorporates a two-phase decision-making structure, time-decaying internal and external influence coefficients, and a dynamic market potential function linked to cumulative sharers. The model also accounts for behavioral and contextual factors such as fan activity, celebrity effect, and information quality. Empirical validation using Weibo movie trailer data demonstrates that the GBM consistently outperforms the classical Bass model as well as two improved variants (PIM and EIM) in terms of both accuracy and explanatory power. The results highlight that internal influence is not only subject to exponential decay but also amplified by social pressure—offering new insights into the dynamics of digital information diffusion.

### Theoretical contributions

This study makes three significant theoretical contributions. First, it introduces a novel diffusion model tailored specifically to product information sharing, extending the Bass model’s relevance from traditional product adoption to dynamic social media environments. Second, it incorporates both interest decay and social pressure into internal influence mechanisms, offering a more comprehensive representation of temporal and behavioral drivers in information diffusion—factors previously underexplored in the literature. Third, by modeling user conversion across heterogeneous information sources (e.g., fan-based vs. non-fan-based channels), the study presents a dynamic formulation of market potential, addressing a critical limitation in prior diffusion models that assume static or exogenous potential.

### Managerial implications

From a practical perspective, our model provides substantial value for marketers, advertisers, and content creators. First, it offers actionable decision support for selecting appropriate publishers and optimizing advertising budgets based on dynamic diffusion patterns. Second, it enables the design of more targeted marketing campaigns by considering the time-decay of user interest and the impact of social pressure. Third, for high-traffic users such as influencers and KOLs, the model helps in determining optimal posting schedules and pricing strategies to maximize engagement and return on investment. Finally, the ability of the model to predict both cumulative and unit-time sharing volumes supports real-time marketing adjustments and long-term strategic planning.

### Methodological implications

Methodologically, this study advances the classical Bass framework by integrating a dual-phase user decision process and time-decaying influence coefficients. Unlike traditional models that treat adoption potential and influence as static, the GBM dynamically models market potential as a function of cumulative sharers and incorporates exponential decay in both internal and external influence factors. This allows the model to better represent the evolving nature of user engagement and attention in social media environments. Moreover, the framework lays a foundation for future extensions that can incorporate additional user-level heterogeneity, feedback loops, or network-based structures.

### Limitations and future research

Despite its contributions, this study has several limitations that warrant further investigation. First, the model assumes a static classification of users into fan and non-fan groups. In reality, user engagement is dynamic, and future research could incorporate user state transitions over time. Second, the current formulation assumes homogeneous social pressure across all users. Future work could integrate social network analysis to account for differences in connectivity, centrality, and community structure. Third, empirical validation is limited to a single platform (Weibo) and a single content type (movie trailers). Broader testing across platforms (e.g., TikTok, Bilibili) and content domains (e.g., e-commerce, education) would enhance generalizability. Finally, the current model focuses on neutral or positive information diffusion. Future studies could explore how negative or emotionally charged content diffuses differently, and how emotional valence influences interest decay and sharing trajectories.

## Data Availability

All data included in this study are available upon request by contact with the corresponding author.
